# Potential risk of gestational diabetes mellitus in females undergoing in vitro fertilization: a pilot study

**DOI:** 10.1186/s40842-024-00164-x

**Published:** 2024-04-10

**Authors:** Yehia Moustafa Ghanem, Yasser El Kassar, May Mohamed Magdy, Mohamed Amara, Noha Gaber Amin

**Affiliations:** 1 Department of Internal Medicine; Unit of Diabetes Lipidology & Metabolism, Faculty of Medicine, Alexandria University, 17 Champollion Street Azarita, Alexandria, Egypt; 2 Department of Obstetrics and Gynecology, Faculty of Medicine, Alexandria University, Alexandria, Egypt; 3 Department of Internal Medicine, Faculty of Medicine, Fayoum University, Fayoum, Egypt

**Keywords:** Gestational diabetes, In vitro fertilization, Pregnancy, OGTT

## Abstract

**Background:**

Most of the cases of hyperglycemia during pregnancy are attributed to gestational diabetes mellitus (GDM) (75–90%). Women diagnosed with GDM are at an increased risk for complications during pregnancy and delivery. This observational prospective study aimed to investigate the potential risk of GDM among Egyptian females following in vitro fertilization (IVF) pregnancies compared to spontaneous pregnancies (SC).

**Methods:**

This prospective cohort study included normoglycemic females without any history of dysglycemia before this conception. Subjects were divided according to the type of conception into two age and BMI-matched groups: (IVF group): 55 pregnant females conceived by IVF, and (SC group) spontaneous pregnancy: 55 pregnant females conceived spontaneously. A one-step oral glucose tolerance test (OGTT) was performed at gestational weeks 20 and 28 for all study subjects.

**Results:**

The incidence of GDM was statistically significantly higher in the IVF group compared to the spontaneous pregnancy (SC) group (20 and 5.5%, respectively), *p* = 0.022 at week 28. On comparing the incidence of GDM on early screening at week 20 in both groups, the incidence of GDM in the IVF group was significantly higher (16.4%) compared to (3.6%) in the spontaneous pregnancy (SC) group, *p* = 0.026.

**Conclusions:**

IVF may have an increased potential risk for GDM. Moreover, the diagnosis of GDM may occur early (week 20), highlighting the need for precise and early screening for GDM in IVF pregnancies.

## Introduction

The number of pregnancies resulting from in vitro fertilization (IVF) is increasing due to the rise in the prevalence of infertility globally. IVF is one of the most effective forms of assisted reproductive technology (ART). IVF-achieved pregnancies are associated with a higher risk for both obstetric and perinatal complications compared to spontaneous pregnancies [1, 2]. Although several studies have reported an increased prevalence of GDM among IVF pregnancies, further investigations are needed to determine the exact time for GDM screening and the management of GDM in IVF pregnancies [3–5].

Gestational diabetes mellitus (GDM) is defined as a condition where a woman without a previous diagnosis of diabetes experiences high blood glucose levels during pregnancy. GDM is one of the most frequent maternal complications during pregnancy, affecting 10-25% of pregnancies worldwide [6]. GDM is the most common type of diabetes during pregnancy, representing 75–90% of cases of diabetes during pregnancy [7].

Factors that increase the risk of GDM include polycystic ovary syndrome, history of GDM in a previous pregnancy, family history of a first-degree relative with type 2 DM, advanced maternal age (over 35 years of age), being overweight, obesity, pre-existing hypertension, smoking during pregnancy, [8] and assisted reproductive technology (ART) treatment such as in vitro fertilization (IVF) [9].

Pregnancy complicated with GDM is associated with adverse acute and long-term consequences for both the mother and the infant [10]. GDM increases the risk of pre-eclampsia, depression, and the necessity of a Caesarean section [11]. Newborns born to mothers with poorly controlled gestational diabetes are at increased risk of macrosomia, hypoglycemia, and jaundice. If untreated, it can also result in stillbirth. In addition, in the long term, children are at a higher risk of being overweight and at a higher risk of developing type 2 diabetes. Furthermore, a long-term follow-up study reported that a high percentage of women with GDM will develop type 2 diabetes [10, 11].

Further multicenter studies are required to confirm the relation between IVF and the prevalence of GDM, whether this is attributed to pre-existing medical conditions or the IVF technique itself. This study aimed to determine the potential risk of GDM among Egyptian females following in vitro fertilization (IVF) pregnancies compared to spontaneous pregnancies. This study also aimed to evaluate the time of diagnosis of GDM in IVF pregnancies compared to spontaneous pregnancies.

### Subjects and methods

#### Study design

- This prospective cohort study design recruited 110 primigravid pregnant females (aged 18-39 at the time of conception). At the outpatient clinic, participants planning for pregnancy and having medical records at the registry of El Shatby Hospital (the central maternity hospital in Alexandria) were invited to participate in the study from January 2021 to September 2021.
- Medical records of the first pre-pregnancy visit and the first ante-natal care visit at week [4–6] of gestation confirmed normoglycemia according to the ADA criteria for the diagnosis of diabetes [12]. Subjects were screened for gestational diabetes at week 20 and week 28 of gestation using the 75 g 2-hour Oral Glucose Tolerance Test (OGTT) as recommended by the International Association of Diabetes in Pregnancy Study Groups (IADPSG) recommendations [13]. GDM was diagnosed based on a single reading above three thresholds: Fasting 92 mg/dL, 1-h 180 mg/dL, and 2-h 153 mg/dL.
- Study subjects were divided into two groups:

**IVF group:** 55 pregnant females who IVF conceived. Our institute’s IVF treatment protocol agreed with the standard international guidelines. The IVF technique applied was gonadotrophin-releasing hormone (GnRH) antagonist in In-vitro-fertilization/Intracytoplasmic sperm injection (IVF/ICSI) cycles [14]. The different infertility underlying etiologies for IVF included maternal structural and mechanical factors (48%), unexplained factors (20%), and male factors (32%).

**Spontaneous pregnancy (SC) group:** 55 pregnant females who conceived spontaneously.

- The ethics committee of Alexandria University approved the study design. The participating study population signed an informed consent before any study-related procedure occurred. The study followed the criteria set by the Declaration of Helsinki. Confidentiality and personal privacy were respected at all levels of the study. Patients felt free to withdraw from the study at any time without any consequences.

#### Exclusion criteria

Subjects with a history of pre-pregnancy existing diabetes, a history of any previous glucose intolerance or pre-diabetes, or a history of GDM in previous pregnancies were excluded from the study. We also excluded subjects with acanthosis nigricans and cases of PCOS according to Rotterdam criteria of diagnosis [15]. Subjects of age more than 39 years at the time of conception, smokers, twin pregnancies, thyroid dysfunction, or any known other chronic condition. Patients taking medications that may affect glucose homeostasis, including corticosteroid medications, anti-inflammatory drugs, antidepressants, bronchodilators, nicotine, thyroid hormones, and growth hormones, were excluded from the study.

## Methods

The following was performed for all the study subjects:

1. **Baseline visit:** At the first antenatal visit at 4-6 weeks of gestation.

A. **History Taking and review of the medical records:**

The medical records of the recruited pregnant females were reviewed using a computerised sheet including all studied data for each subject to exclude undiagnosed pre-existing diabetes and check the HbA1c at the confirmation of pregnancy. A thorough history of any chronic diseases, drug history, family history of diabetes, hypertension, and other medical history was taken.

B. **Medical records were checked to report pre-pregnancy data, including.**

1. Body weight, height, and body mass index (BMI) (kg/meter^2^)
2. Vital signs: Blood pressure measurement and pulse examination.

C. **Laboratory assessment:**

Participants were referred to the same central laboratory after 8-10 hours of overnight fasting. Blood samples were collected to assess the fasting plasma glucose level (FPG), plasma glucose level after 2 hours (2-hour PP) of 75 g anhydrous glucose load, and HbA1c to exclude pre-pregnancy existing diabetes at the first ant-natal visit.

2- **Evaluation At 20 weeks of gestation for GDM using one-step OGTT.**
3- **Evaluation At 28 weeks of gestation for GDM using one-step OGTT** [16].

### Sample collection and preparation

- Venous blood samples were obtained for all lab tests.
- OGTT was assessed by 8-10 hour fasting of a patient all night; the sample was collected in the morning in a grey top (Na fluoride/K oxalate) tube and centrifuged at 1100-2000 g for a minimum of 10 minutes, 75 g of glucose given immediately and after 1 hour and two-hour sample were collected again.
- HbA1c was collected in a vacutainer tube containing Na2-EDTA and centrifuged (3000 rpm) for serum preparation.

### Statistical analysis

All statistical analyses were performed using the SPSS software (version 20.0; IBM Corporation, Armonk, NY, USA). Continuous data were presented as mean ± SD, and categorical data as numbers and percentages. We used the student’s *t*-test to compare the normally distributed quantitative variables between the two main groups. The chi-square test was used to compare the categorical variables between the main groups, given that Fisher Exact and Monte-Carlo Exact tests were used instead in case of violating the chi-square test assumptions. The crude odds ratio with its 95% confidence interval was estimated for testing the risk of developing GDM among the IVF and the spontaneous pregnancy groups. A *P* value < 0.05 was considered significant.

## Results

Table 1 shows the study population’s baseline characteristics.

**Table 1:** The baseline characteristics of the study population

	IVF group (***n*** = 55)		SC Group (***n*** = 55)		***P***-value
	No	%	No	%	
**Age at the time of conception (years)**	37.54 ± 1.24		37.83 ± 1.08		0.192^a^
**Pre- Pregnancy BMI (kg/m**^**2**^**)**					
Normal (18.5 – 24.9)	5	9.1	4	7.3	0.112^c^
Overweight (25– 29.9)	31	56.4	21	38.2	
Obese (≥ 30)	19	34.5	30	54.5	
**Pre-pregnancy systolic BP (mmHg)**	112.91 ± 12.05		116.91 ± 11.16		0.074^a^
**Pre-pregnancy diastolic BP (mmHg)**	71.09 ± 10.48		72.27 ± 10.71		0.560^a^
**Family history of diabetes**	19	34.5	30	54.5	0.035*^b^

^a^: *p-value* (< 0.05) was considered significant using the student *t*-test

^b^: *p-value* (< 0.05) was considered significant using the Chi-Square test

^c^: *p-value* (< 0.05) was considered significant using the Monte- Carlo Exact test

The baseline comparison between the two studied groups showed no significant difference regarding age, BMI, systolic blood pressure, and diastolic blood pressure. However, the presence of a positive family history of diabetes was statistically significantly higher among the SC group compared to the IVF pregnancy group. It was also noted that only 9.1 and 7.3% had normal BMI in the IVF group and the SC Group, respectively, while most of both groups were either overweight or obese.

### Parameters of glycemic profile

A 75 g OGTT to screen for GDM was done twice, at weeks 20 and 28 of pregnancy. At week 20 of pregnancy, **16.4%** in the IVF group had GDM compared to **3.6%** in the SC group, which was statistically significant with ***p*-value = 0.026.** At week 28 of pregnancy, 20% in the IVF group had GDM compared to 5.5% in the SC group, which was statistically significant with ***p*-value** = **0.022,** as shown in Table 2.

**Table 2:** Comparing the Incidence of GDM among the IVF & and spontaneous pregnancy groups

GDM	IVF group (n = 55)		SC Group (***n*** = 55)		χ^**2**^	***p***	Crude OR	95% C. I for the Crude OR
	No.	%	No.	%				L.L – U.L
**20 Weeks**								
No	46	83.6	53	94.4	4.949^*^	0.026^*^	15.185	(1.06 – 25.23)
Yes	9	16.4	2	3.6				
**28 Weeks**								
No	44	80.0	52	94.5	5.238^*^	0.022^*^	14.333	(1.14 – 16.52)
Yes	11	20.0	3	5.5				

^*^P-value (< 0.05) was considered significant using the Chi-Square test

### Comparing the incidence of GDM among the IVF & and the spontaneous pregnancy groups

IVF pregnancies had a **five-fold** increased risk for GDM compared to the SC pregnancies at week 20 of pregnancy, and **four-fold** increased risks at 28 weeks of gestation, as shown in Table 2.

## Discussion

The present work aimed to assess the potential risk of GDM among Egyptian females conceived by IVF procedures (IVF) compared to spontaneous (SC) pregnancies. This study also aimed to investigate the time for screening of GDM in IVF pregnancies.

In our study, the in vitro fertilization (IVF) and SC pregnancy groups were age- and BMI-matched. The mean pre-pregnancy BMI was 29.74 ± 5.33 kg/m^2^ in the IVF group and 32.02 ± 6.17 kg/m^2^ in the SC pregnancies group, with a non-significant difference between both groups (*p* = 0.068). Overweight and obesity are major risk factors for GDM. The prevalence of GDM globally is increasing parallel to the rising surge of obesity in the reproductive age among females [17]. Kouhkan et al. [18] demonstrated that overweight and obese women had roughly three- and five-fold increases in the odds of developing GDM, respectively. Provost et al. [19] reported a higher rate of being overweight (22.9%) and obese (17.8%) among women undergoing ART. Torloni et al. [14], in a meta-analysis of 70 studies, reported that the risk of developing GDM in overweight and obese women in spontaneous pregnancies was nearly 2- and 4-fold higher in comparison to women with a normal BMI. Moreover, they demonstrated that in women with BMI > 25 kg/m^2^, adding 1 kg/m^2^ BMI increased the risk of developing GDM by approximately 0.92%.

Regarding the potential risk of GDM following ART, Xiong et al. reported a linear positive association between pre-pregnancy body weight and the risk of GDM in a population-based cohort study. These findings support the recommendations for pre-pregnancy weight intervention, especially before starting ART procedures [20].

Our results demonstrated a statistically significant higher incidence of GDM in the IVF group compared to the spontaneous pregnancy group at 20 and 28 weeks of pregnancy, *p* = 0.026 and 0.022, respectively. Also, an increased risk of developing GDM in the IVF group than in the spontaneous pregnancy group at 20 weeks (OR 5.185, 95% CI: 1.06 – 25.23), at 28 weeks (OR 4.333, 95% CI: 1.14 – 16.52). This was in harmony with the results of the Pandey et al. meta-analysis [21], which reported that the relative risk (95% CI) of having gestational diabetes was 1.48 (1.33–1.66) in IVF conceptions when compared with spontaneous conceptions with an absolute increased risk (95% CI) of 1% (1–1%). Cai et al. [22] showed that IVF pregnancies were related to a higher rate of GDM alongside raised fasting and 2-hour OGTT blood glucose levels in the late second trimester, particularly in overweight and obese mothers. Results of the Mohammadi et al. metanalysis [23] demonstrated a significant increase in GDM among women who conceived by ART in comparison to those who conceived spontaneously (pooled relative risk = 1.51, 95% confidence interval = 1.18–1.93). In agreement with our observations, Thomakos et al. reported that 37.6% of the IVF pregnancy group was diagnosed with GDM before the 24th week of gestation [24].

Also, a meta-analysis study by Bosdou et al. [4], which included 63,760 females who got pregnant after ART (GDM was present in 4776) and 1,870,734 females who got pregnant spontaneously (GDM in 158,526), revealed a higher risk of GDM after ART versus Spontaneous conceptions (RR 1.95, 95% CI 1.56–2.44). Our observations emphasise the importance of recognising IVF as an important risk factor for GDM, and raising awareness among clinicians would help better prevent GDM in the future. Moreover, current guidelines do not have specific recommendations regarding screening and management for GDM in IVF pregnancies; our data may be used to further evaluate the benefit of early screening for GDM (before 24-28 weeks of gestation) in IVF pregnancies [13].

Many females undergoing ART conceptions have significant risk factors for GDM, such as advanced maternal age, obesity, multiple pregnancies and polycystic ovary syndrome (PCOS), suggesting a potential relationship between GDM and ART [25]. Another explanation may be attributed to the high dose of gestational hormones administered with IVF techniques, which may precipitate metabolic derangements, insulin resistance, and glucose intolerance. However, further studies are required to establish these effects [21, 26].

Our study has some limitations, mainly because this was performed in one centre; thus, the results may not apply to other populations. A significant limitation of our study was the lack of funding to investigate a larger sample size, as the post-hoc power analysis based on the given results of a total sample size of 110 (55 per group) is estimated to be 70% given that GDM was statistically significantly higher in IVF group compared to spontaneous pregnancy group (20 and 5.5% respectively). Thus, we recommend further multicentric research, recruiting a larger sample size. This study aimed to test the potential increased incidence of GDM among IVF-induced pregnancies. Thus, further research is recommended to investigate various risk factors implicated.

## Conclusion

GDM is a common health problem in our community. The risk of GDM is increased by five-fold among IVF-induced pregnancies compared to spontaneous pregnancies. Our observations shed light on the recognition of IVF as a risk factor for GDM and that early screening for GDM in IVF pregnancies may help the early diagnosis of GDM.

## Data Availability

The datasets used and analysed during the current study are available from the corresponding author upon reasonable request.

## Abbreviations

GDM: Gestational diabetes
IVF: In vitro fertilization
ART: Assisted reproductive technology
PCOS: Polycystic ovarian syndrome
OGTT: Oral glucose tolerance test
T2DM: type 2 diabetes
HbA1c: Glycosylated hemoglobin
BMI: Body mass index
